# Association between humidifier disinfectant use duration and lung cancer development in Korea

**DOI:** 10.4178/epih.e2025023

**Published:** 2025-05-02

**Authors:** Sungchan Kang, Jeong-In Hwang, Su Hwan Kim, Hyungryul Lim, Dong-wook Lee, Woojoo Lee, Jong Hun Kim, Sol Yu, Jungyun Lim, Younghee Kim, Kyoung-Nam Kim

**Affiliations:** 1 Department of Public Health Sciences, Graduate School of Public Health, Seoul National University, Seoul, Korea; 2 Department of Preventive Medicine, Yonsei University College of Medicine, Seoul, Korea; 3 Department of Information Statistics, Gyeongsang National University, Jinju, Korea; 4 Department of Preventive Medicine and Public Health, Ajou University School of Medicine, Suwon, Korea; 5 Department of Occupational and Environmental Medicine, Inha University Hospital, Incheon, Korea; 6 Department of Social and Preventive Medicine, Sungkyunkwan University School of Medicine, Suwon, Korea; 7 Humidifier Disinfectant Health Center, National Institute of Environmental Research, Incheon, Korea

**Keywords:** Lung neoplasms, Humidifier disinfectants, Cox proportional hazards model, Polyhexamethylene guanidine phosphate

## Abstract

**OBJECTIVES:**

This study was conducted to assess the association between the duration of humidifier disinfectant use and lung cancer development.

**METHODS:**

We analyzed data from 3,605 applicants registered for compensation from the Korean government due to health conditions related to humidifier disinfectant exposure. Among these individuals, 121 were diagnosed with lung cancer at least 4 years after their initial exposure (through December 2021). Hazard ratios (HRs) and 95% confidence intervals (CIs) for lung cancer incidence were estimated according to the duration of disinfectant use using Cox proportional hazards models.

**RESULTS:**

Compared with <5 months of use, the HRs for lung cancer were 1.81 (95% CI, 0.41 to 7.97) for 5-14 months, 2.45 (95% CI, 0.58 to 10.41) for 15-29 months, and 4.61 (95% CI, 1.12 to 18.91) for ≥30 months. Using never smokers with <15 months of use as the reference category, the HRs were 2.97 (95% CI, 1.34 to 6.56) for never smokers with ≥15 months of use, 2.73 (95% CI, 0.94 to 7.95) for current or former smokers with <15 months of use, and 4.74 (95% CI, 1.94 to 11.61) for current or former smokers with ≥15 months of use.

**CONCLUSIONS:**

Our study provides some of the first robust epidemiological evidence that prolonged humidifier disinfectant use contributes to lung cancer development. Future studies—particularly those including unexposed populations—are needed to confirm these findings.

## GRAPHICAL ABSTRACT

[Fig f4-epih-47-e2025023]

## Key Message

• There is a quantitative association between prolonged humidifier disinfectants use and the risk of lung cancer incidence.

• Individuals reported they used humidifier disinfectants for more than 30 months had more than four times higher risk of lung cancer than those of individuals who reported they used humidifier disinfectants for less than five months.

## INTRODUCTION

Humidifier disinfectants, added to water in home humidifiers to prevent microbial growth and scale formation, were first introduced in the Korea in 1994. These over-the-counter biocides, available in 41 products [1], were widely used until 2011 [2]. Notably, unexplained fatal lung injuries were reported in children in 2006 [3], with similar cases emerging in adults in 2011 [2]. An epidemiological investigation by the Korea Disease Control and Prevention Agency identified a link between humidifier disinfectant use and lung injuries, including interstitial pneumonitis and extensive pulmonary fibrosis [4]. Consequently, the Korean government banned the sale of these disinfectants and mandated their recall in 2011 [5].

Humidifier disinfectants contain various agents, including polyhexamethylene guanidine (PHMG; CAS No. 31961-54-3), oligo-(2-(2-ethoxy)-ethoxyethyl) guanidine (CAS No. 374572-91-5), and a combination of chloromethylisothiazolinone (CAS No. 26172-55-5) and methylisothiazolinone (CAS No. 2682-20-4). Among them, PHMG—a major component of these disinfectants that is commonly found in household products such as shampoos and moist towelettes [6,7]—has been designated by the International Agency for Research on Cancer as a high-priority agent for carcinogenicity evaluation [8]. Although animal studies increasingly demonstrate that PHMG exposure promotes lung cancer development [9], epidemiological investigations into the link between humidifier disinfectant use and lung cancer remain limited.

Therefore, this study aimed to evaluate the association between the duration of humidifier disinfectant use and the development of lung cancer among individuals who participated in Korean government surveys on humidifier disinfectant-related harm. To our knowledge, this represents one of the first epidemiological studies to investigate the potential impact of humidifier disinfectants on lung cancer. We anticipate that our findings will provide critical evidence establishing the carcinogenic potential of biocides used in these products.

## MATERIALS AND METHODS

### Study population

This study utilized data from Korean government surveys of individuals with a history of humidifier disinfectant use who filed compensation claims for associated health damage. Initially, only a few pre-specified diseases were eligible for compensation, among which lung cancer was not included. However, following a legal amendment in 2020, this limitation on diseases designated as related to humidifier disinfectant exposure was lifted. In most lung cancer cases (115 of 121 applicants), exposure assessments—including the duration of disinfectant use—were conducted before diagnosis.

Data were collected from claimants by the Korea Environmental Industry & Technology Institute [10], with the first survey initiated in July 2013. For this analysis, we included data from all surveys conducted through December 2021, the most recent available. Applicants whose data collection was complete were reviewed and registered for compensation by the Committee for the Determination of Humidifier Disinfectant-related Health Effects [10] under the “Special Act on Remedy for Damage Caused by Humidifier Disinfectants.” Trained occupational and environmental health nurses or industrial hygienists conducted standardized interviews to document humidifier disinfectant exposure, medical history, socio-demographic characteristics, lifestyle factors, household environment, and occupational exposures [11]. This study did not incorporate data from external sources such as the National Health Insurance Service, National Cancer Registry, or Death Registry.

**Figure 1.**
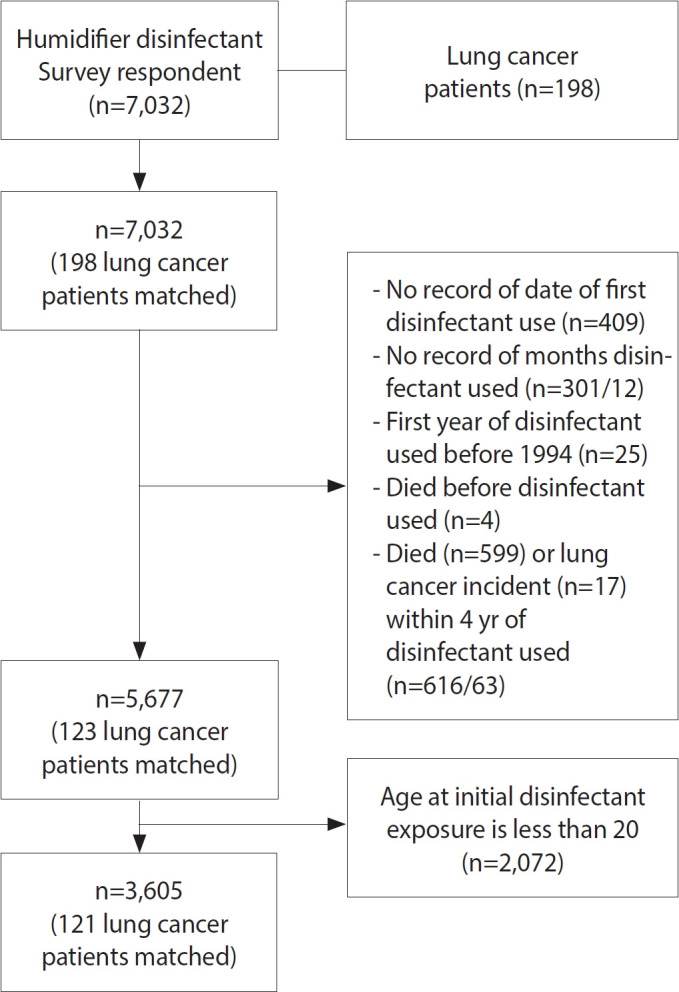
Flowchart of the study population.

Among 7,032 applicants, 6,834 had not developed lung cancer by December 31, 2021, while 198 had developed lung cancer by that date. Of the 6,834 applicants without lung cancer, we excluded 409 individuals who lacked information on the date of initial humidifier disinfectant use, 289 who were missing information on duration of use, 25 who reported use before 1994, 4 whose recorded date of death preceded their initial use, 553 who died within 4 years of initial use, and 2,070 whose first exposure occurred at age 19 or younger. From the 198 applicants who developed lung cancer by December 31, 2021, we excluded 12 who lacked information on duration of use, 63 who were diagnosed with lung cancer or died within 4 years of initial use, and 2 who were first exposed at age 19 or younger. The final sample comprised 3,605 individuals, of whom 121 were diagnosed with lung cancer and 3,484 were not diagnosed (Figure 1).

### Exposure to humidifier disinfectants

Humidifier disinfectant exposure was assessed using a standardized interview containing a series of exposure-related questionnaires. Among the investigated exposure variables—including duration of use, exposure history, product name, usage behavior, and distance from the humidifier—we selected duration of use (in months) as the main exposure variable because it had the fewest missing values and outliers. Consistent with a previous study [12], we then categorized duration into 4 groups: <5 months, 5-14 months, 15-29 months, and ≥30 months.

### Reported health conditions

After obtaining consent for the use of personal information, occupational and environmental medicine specialists commissioned by the Committee for the Determination of Humidifier Disinfectant-related Health Effects reviewed the applicants’ medical records to confirm lung cancer diagnoses and to collect detailed information on the date of diagnosis, cancer stage, and prognosis/outcomes, including death.

We defined lung cancer cases as those first diagnosed at least 4 years after initial exposure to humidifier disinfectants [13,14]. We also repeated the analyses using extended latency periods of 6 years and 8 years.

### Statistical analysis

**Figure 2.**
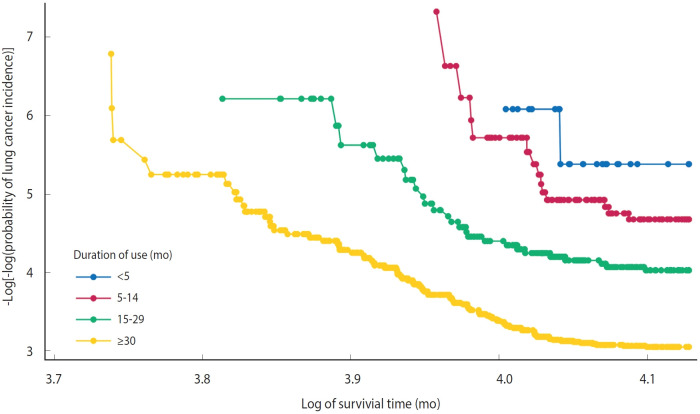
Log-log survival plot depicting the association between the duration of humidifier disinfectant use and lung cancer risk.

Hazard ratios (HRs) and 95% confidence intervals (CIs) for lung cancer occurrence by duration of humidifier disinfectant use were estimated using Cox proportional hazards models. The baseline for the analyses was defined as the date of first humidifier disinfectant use, and follow-up continued until the earliest of: December 31, 2021; the date of lung cancer diagnosis (most recent case: April 8, 2020); or death. Individuals were censored at death if it occurred before December 31, 2021. We evaluated the proportional hazards assumption and confirmed that it was not violated (p=0.37; Figure 2).

The Cox regression models were adjusted for sex, age at initial exposure (20-39, 40-49, or ≥50 years), education level (middle school or lower, high school, or college or higher), tobacco smoking status (never smoked, current, or former smoker), and distance from the humidifier (<0.5, 0.5-0.9, 1.0-1.9 or ≥2.0 m).

Because humidifier disinfectant exposure and tobacco smoking are both major risk factors for lung cancer and may act synergistically [15], we constructed a joint exposure variable combining disinfectant use duration (<15 vs. ≥15 months) and smoking status (never, current, or former smoker). We then applied similar Cox regression models, adjusted for the covariates detailed above, to assess this joint association with lung cancer, using never smokers with <15 months of disinfectant use as the reference group. The relative excess risk due to interaction (RERI) was estimated using the delta method [16].

Stratified analyses were conducted by sex, age at initial exposure (20-49 vs. ≥50 years), and education level (high school or lower vs. college or higher) to assess potential heterogeneity in the association between disinfectant use duration and lung cancer development. Stratification by disinfectant ingredient and by smoking status was not performed because limited sample sizes precluded stable estimates.

To assess the sensitivity and robustness of our findings, we repeated the analyses using latency periods of 6 years and 8 years instead of 4 years.

All statistical analyses were performed using Stata version 18.0 MP (StataCorp., College Station, TX, USA), with a 2-sided p-value of less than 0.05 considered to indicate statistical significance.

### Ethics statement

The study protocol was reviewed and approved by the Institutional Review Board of Severance Hospital (IRB No. 4-2024-11194). Written informed consent was obtained from all compensation applicants or, for those under 19 years of age, from their parents.

## RESULTS

### Study population

Table 1 presents the general characteristics of the study population (n=3,605), stratified by lung cancer status. Overall, 47.4% (n=1,707) were male, 52.5% (n=1,892) were first exposed to humidifier disinfectants between the ages of 20 years and 39 years, 52.2% (n=1,882) had an education level of college or higher, and 66.2% (n=2,386) had never smoked. The proportion of individuals who developed lung cancer was greater among those with a middle school education or lower and among current or former smokers, relative to their counterparts with higher education or who never smoked.

### Distribution of lung cancer development by exposure characteristics

The proportion of humidifier disinfectant use for at least 30 months was substantially higher among individuals diagnosed with lung cancer compared to those without this diagnosis (67.9 vs. 41.3%). In addition, those with lung cancer diagnoses tended to report shorter distances from the humidifier, although this difference was modest (Table 2).

The average duration of humidifier disinfectant use was longer among applicants diagnosed with lung cancer than among those without such a diagnosis. The mean±standard deviation duration of use was 53.4±37.9 months in the lung cancer group versus 33.5±31.9 months in the non-lung cancer group. The median durations were 45 months and 24 months for the lung cancer and non-lung cancer groups, respectively (Table 3).

### Association between the duration of humidifier disinfectant use and lung cancer risk

The analysis included 53,966.2 person-years of observation, during which 121 incident lung cancer cases were identified. Compared with <5 months of humidifier disinfectant use, the HRs for lung cancer incidence were 1.81 (95% CI, 0.41 to 7.97) for 5-14 months, 2.45 (95% CI, 0.58 to 10.41) for 15-29 months, and 4.61 (95% CI, 1.12 to 18.91) for ≥30 months. When duration of use was modeled as a continuous variable in the same analytical model, each additional month of use was associated with an HR of 1.01 (95% CI, 1.00 to 1.01) for lung cancer incidence (Table 4).

In the joint exposure analyses combining humidifier disinfectant use duration (<15 vs. ≥15 months) and smoking status (never vs. current/former smoker), and using never smokers with <15 months of use as the reference, the HRs of lung cancer incidence were 2.97 (95% CI, 1.34 to 6.56) for never smokers with ≥15 months of use, 2.73 (95% CI, 0.94 to 7.95) for current or former smokers with <15 months of use, and 4.74 (95% CI, 1.94 to 11.61) for current or former smokers with ≥15 months of use. We found no evidence of interaction between the duration of humidifier disinfectant use and smoking status (RERI, 0.04; 95% CI, -2.57 to 2.65).

**Figure 3.**
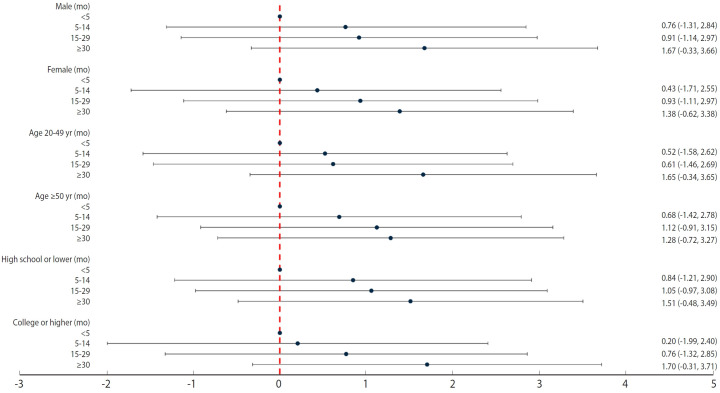
Associations between the duration of humidifier disinfectant use and lung cancer risk, stratified by sex, age at initial exposure (20-49 vs. ≥50 years), and education level (high school or lower vs. college or higher). The results are presented as natural log-transformed hazard ratios with corresponding log-transformed 95% confidence intervals.

In the analyses stratified by sex, age at initial exposure (20-49 vs. ≥50 years), and education level (high school or lower vs. college or higher), we observed consistent, monotonic dose-response relationships between disinfectant use duration and lung cancer risk across all strata. However, no statistically significant associations were detected within a stratum, likely due to the small sample sizes. Furthermore, the CIs overlapped across groups, indicating no appreciable heterogeneity across these stratifying variables. Nevertheless, point estimates were relatively high among maled, individuals first exposed to humidifier disinfectant at 20-49 years of age, and those with a college education or higher (Figure 3, Supplementary Material 1).

In the sensitivity analyses using extended latency periods of 6 years and 8 years, we observed similar monotonic associations between disinfectant use duration and lung cancer risk, although the strength of these associations attenuated with longer latency (Supplementary Material 2).

## DISCUSSION

In this study, prolonged use of humidifier disinfectants was associated with a dose-response increase in lung cancer risk, even after adjusting for key covariates such as tobacco smoking. Specifically, compared with <5 months of use, the HR for lung cancer was significantly greater for ≥30 months, with the point estimate exceeding 4. These results provide robust epidemiological evidence for the role of humidifier disinfectants in lung cancer development, consistent with findings from prior animal studies.

Although lung cancer incidence was higher in our study population compared to the general population, previously established risk factors for lung cancer (e.g., lower educational attainment and tobacco smoking) were similarly associated with increased lung cancer risk in this study (Table 1). Moreover, we observed a dose-response relationship between humidifier disinfectant exposure and lung cancer (Tables 2 and 3, Figures 2 and 3). Collectively, these findings can be interpreted as supporting the association between humidifier disinfectant use and lung cancer risk, an association unlikely to be explained solely by selection bias within this study population.

Several animal studies have demonstrated that PHMG—a major ingredient in humidifier disinfectants—promotes lung carcinogenesis. For example, exposure to PHMG phosphate (PHMG-p), a common form of PHMG, increased the risk of lung cancer even after the cessation of respiratory exposure, yielding a 73.7% incidence rate in rats at high doses. That study also reported that PHMG-p exposure induced lung inflammation, fibrosis, precancerous lesions, and somatic mutations in lung cancer-related genes such as *TP53* [17]. In mice, intratracheal administration of a PHMG solution resulted in bronchoalveolar adenomas in 50% of animals within 6-8 weeks, along with genetic alterations associated with lung cancer [9]. Similarly, a single intratracheal instillation of PHMG induced lung carcinoma and progressive fibrosis in rats [18,19].

The observed association between humidifier disinfectant use duration and lung cancer development may in part reflect the reported link between humidifier disinfectant exposure and interstitial lung disease (ILD). ILD is strongly associated with lung cancer [20] due to overlapping biological processes, such as fibrogenesis and carcinogenesis, as well as shared risk factors [21,22]. Epidemiological studies have revealed a significant relationship between humidifier disinfectant exposure and the risk of ILD, along with ILD-related features like ground-glass opacities, centrilobular fibrosis, and air leakage [23]. Similarly, an animal study in rats demonstrated that humidifier disinfectant exposure induces lung fibrosis and inflammatory responses resembling ILD-like pathology [24]. Together, these findings further support the link between disinfectant use duration and lung cancer development observed in this study.

Within the aggregate exposure pathway-adverse outcome pathway (AEP-AOP) framework, humidifier disinfectant chemicals such as PHMG have been shown to induce a cascade of events, including oxidative stress, inflammation, epithelial damage, and necrosis, that culminate in severe clinical outcomes like ILD and lung cancer [25]. PHMG-p drives lung carcinogenesis primarily through non-genotoxic mechanisms, including chronic inflammation, apoptosis, and secondary DNA damage [26]. Furthermore, prolonged PHMG-p exposure alters the expression of lung cancer-associated genes in human pulmonary alveolar epithelial cells, underscoring its carcinogenic potential [27].

We observed no evidence of interaction between the duration of humidifier disinfectant use and tobacco smoking for lung cancer risk. To our knowledge, this is the first study to examine this potential interaction, and our finding thus warrants validation in future research. However, the independent associations of disinfectant exposure and smoking with lung cancer suggest distinct biological pathways for these risk factors. Given the substantial overlap between the previously described AEP-AOP and the carcinogenic pathways of tobacco smoke, further mechanistic studies are needed to delineate the pathways associated with humidifier disinfectant exposure.

This study had several limitations. First, the study population consisted of individuals exposed to humidifier disinfectants who reported at least 1 health condition when registering for government compensation, raising concerns about selection bias and limiting generalizability. However, lung cancer was only recently recognized as a compensable disease for claims related to humidifier disinfectant exposure. Thus, participants were not enrolled specifically because of a lung cancer diagnosis. Furthermore, animal studies demonstrating a causal relationship between humidifier disinfectant exposure and lung cancer suggest that our findings cannot be entirely explained by selection or collider stratification bias. Future epidemiological investigations using diverse populations, study designs, and analytic methods robust to selection bias—such as propensity score techniques and instrumental variable approaches—are warranted to confirm our results. Second, exposure assessment relied on self-reported data, introducing the possibility of recall bias. Although well-structured interviews with logical and repetitive questioning were conducted to mitigate this concern, differential misclassification based on health status (e.g., symptom severity) may still have inflated associations. Third, because of the long latency between exposure and lung cancer onset, cancer may have manifested only in a subset of at-risk individuals, despite a follow-up period of over 10 years. Consequently, the incidence may continue to rise, particularly among those exposed to humidifier disinfectants at younger ages.

Nonetheless, the present study has several strengths. First, it is among the first epidemiological investigations to offer robust evidence linking prolonged humidifier disinfectant use to an elevated risk of lung cancer. Although animal studies had previously supported this association, epidemiological research remains limited. Second, lung cancer diagnoses were confirmed by physicians through medical record review, minimizing the risk of outcome misclassification and ensuring that the results reflect verified diagnoses. Third, this study was adjusted for key lung cancer risk factors, including smoking status, and also evaluated the potential synergistic interaction between disinfectant use duration and smoking in lung cancer development.

In conclusion, prolonged use of humidifier disinfectants was associated with a dose-dependent increase in lung cancer risk. Compared with <5 months of use, individuals with ≥30 months of exposure had a HR exceeding 4 for lung cancer. These findings provide compelling epidemiological evidence supporting the role of prolonged humidifier disinfectant use in the development of lung cancer. However, further research—including studies involving unexposed populations—is needed to validate these results.

## Figures and Tables

**Figure f4-epih-47-e2025023:**
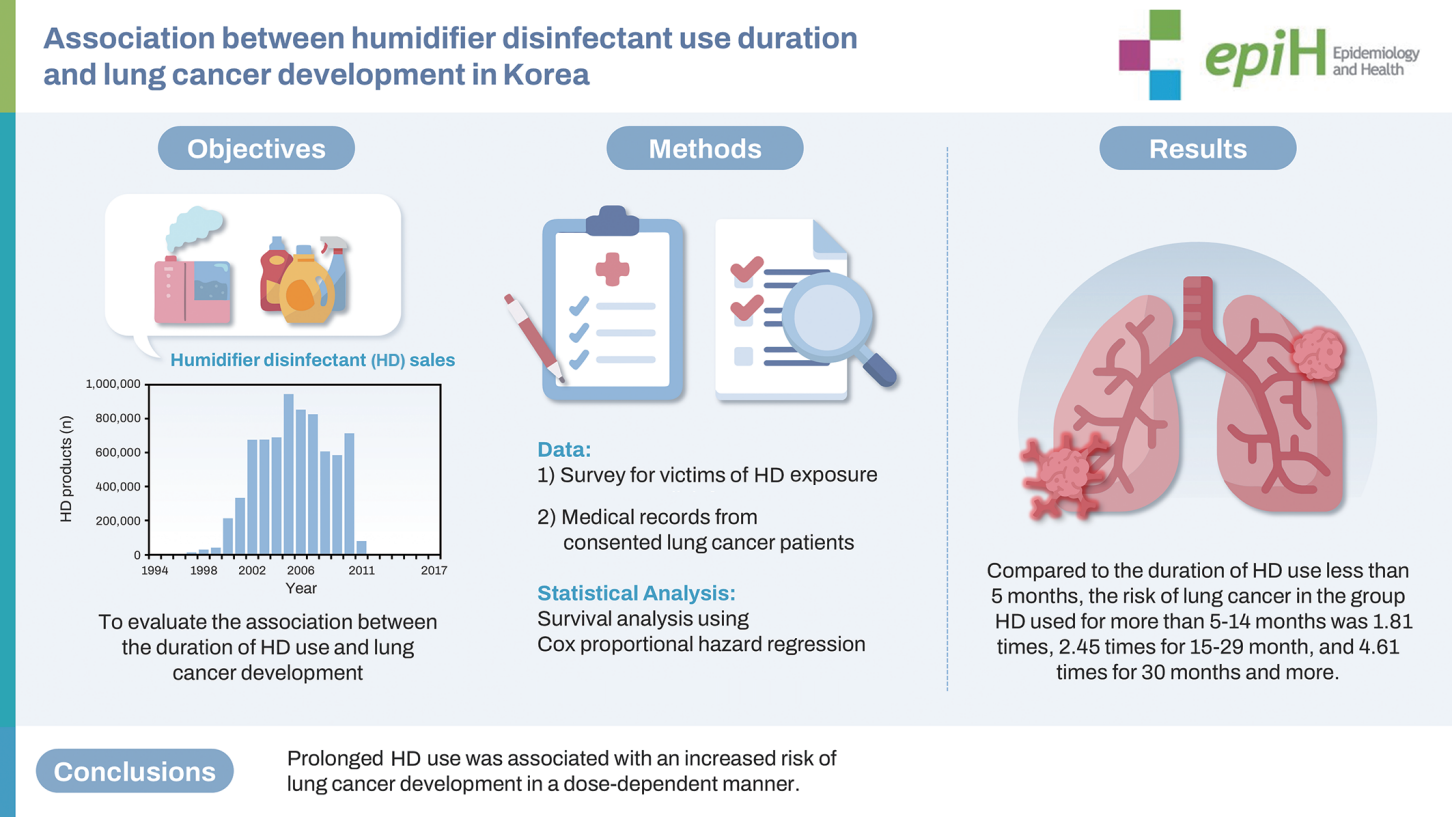


**Table 1.** Characteristics of the study population

Characteristics	Total	Lung cancer	No lung cancer	p-value^1^
Overall	3,605	121 (3.4)	3,484 (96.6)	
Sex				0.21
Male	1,707	64 (3.7)	1,643 (96.3)	
Female	1,898	57 (2.5)	1,841 (97.5)	
Age at initial exposure (yr)				0.14
20-39	1,892	37 (2.0)	1,855 (98.0)	
40-49	553	26 (4.7)	527 (95.3)	
≥50	1,160	58 (5.0)	1,102 (95.0)	
Education level				<0.01
Middle school or lower	689	35 (5.1)	654 (94.9)	
High school	1,034	39 (3.8)	995 (96.2)	
College or higher	1,882	47 (2.5)	1,835 (97.5)	
Tobacco smoking status				<0.01
Never smoker	2,386	66 (2.8)	2,320 (97.2)	
Current or former smoker	1,219	55 (4.5)	1,164 (95.5)	
Survival status (as of 2021)				<0.01
Survived	2,858	51 (1.8)	2,807 (98.2)	
Deceased	747	70 (9.4)	677 (90.6)	

Values are presented as number or number (%).

1 Using chi-square tests for sex and survival status and Cochran–Armitage trend tests for all other variables.

**Table 2.** Distribution of lung cancer development by exposure characteristics

Variables	Total (n=3,605)	Lung cancer (n=121)	No lung cancer (n=3,484)	p-value^1^
Duration of humidifier disinfectant use (mo)				<0.01
<5	240	2 (0.8)	238 (99.2)	
5-14	909	14 (1.5)	895 (98.5)	
15-29	934	23 (2.5)	911 (97.5)	
≥30	1,522	82 (5.4)	1,440 (94.6)	
Distance from humidifier (m)				0.02
<0.5	1,173	45 (3.8)	1,128 (96.2)	
0.5-0.9	1,304	54 (4.1)	1,250 (95.9)	
1.0-1.9	839	16 (1.9)	823 (98.1)	
≥2.0	276	6 (2.2)	270 (97.8)	

Values are presented as number or number (%).

1 Using Cochran–Armitage trend tests.

**Table 3.** Distribution of humidifier disinfectant use duration among the study population

Humidifier disinfectant usage period (mo)	Mean±SD	Min	Median	Max
Total	34.2±32.3	0.1	24	221
Lung cancer	53.4±37.9	1.0	45	180
No lung cancer	33.5±31.9	0.1	24	221

SD, standard deviation; Min, minimum; Max, maximum.

**Table 4.** HRs and 95% CIs for lung cancer occurrence by duration of humidifier disinfectant use^1^

Duration (mo)	n	Person-years	Lung cancer	Mean±SD (yr)^2^	HR (95% CI)
Categorical variable					
<5	240	3,042.5	2	52.4±13.4	1.00 (reference)
5-14	909	12,215.4	14	53.1±12.8	1.81 (0.41, 7.97)
15-29	934	13,518.7	23	55.3±13.2	2.45 (0.58, 10.41)
≥30	1,522	25,189.4	82	59.4±13.2	4.61 (1.12, 18.91)
Continuous variable					
Per-1	3,605	53,966.2	121	56.3±13.4	1.01 (1.00, 1.01)

HR, hazard ratio; CI, confidence interval; SD, standard deviation.

1 The results were analyzed using Cox proportional hazards models adjusted for sex, age at initial exposure, education level, tobacco smoking status, and distance from the humidifier.

2 Mean age at the end of follow-up.
