# Supplementary material for: Association between humidifier disinfectant use duration and lung cancer development in Korea
Source: Epidemiol Health. 2025 May 2;47:e2025023. doi: 10.4178/epih.e2025023 (PMC12425697; doi:10.4178/epih.e2025023)
Supplement: Supplementary Material 1. [file epih-47-e2025023-Supplementary-1.docx]

*Supplementary Material 1*

**Association between humidifier disinfectant use duration and lung cancer development in the Republic of Korea**

Sungchan Kang, Jeong-In Hwang**,** Su Hwan Kim, Hyungryul Lim, Dong-wook Lee, Woojoo Lee, Jong Hun Kim, Sol Yu, Jungyun Lim, Younghee Kim, Kyoung-Nam Kim

**Table of Contents**

**Figure S1.** Associations between the duration of humidifier disinfectant use and the risk of lung cancer, stratified by sex, age at initial exposure (20–49 years vs ≥ 50 years), and educational level (high school or lower vs. college or higher). The results are presented as natural log-transformed hazard ratios along with their natural log-transformed 95% confidence intervals.

**Table S1.** Hazard ratios and 95% confidence intervals for lung cancer occurrence based on duration of humidifier disinfectant use, stratified by sex, age at initial exposure, and educational level


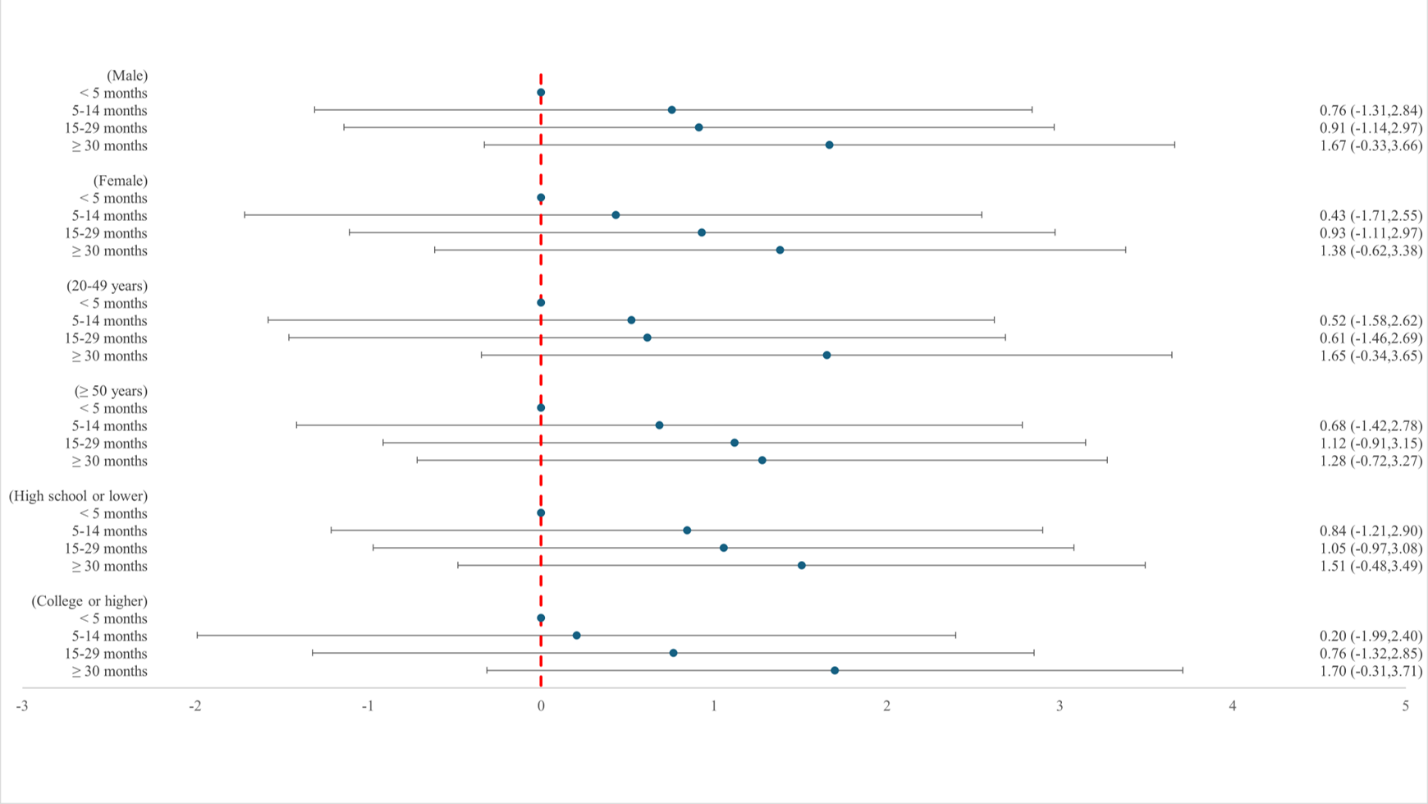


**Figure S1.** Associations between the duration of humidifier disinfectant use and the risk of lung cancer, stratified by sex, age at initial exposure (20–49 years vs ≥ 50 years), and educational level (high school or lower vs. college or higher). The results are presented as natural log-transformed hazard ratios along with their natural log-transformed 95% confidence intervals.

**Table S1.** Hazard ratios and 95% confidence intervals for lung cancer occurrence based on duration of humidifier disinfectant use, stratified by sex, age at initial exposure, and educational level

|  | *n* | Person-year | HR | 95% CI | *p*-value for trend |
| --- | --- | --- | --- | --- | --- |
| Male |  |  |  |  | < 0.01 |
| < 5 months | 217 | 1,310.1 | Ref. | Ref. |  |
| 5–14 months | 804 | 5,651.8 | 2.13 | 0.27, 17.10 |  |
| 15–29 months | 797 | 6,222.9 | 2.49 | 0.32, 19.41 |  |
| ≥ 30 months | 1106 | 12,207.8 | 5.30 | 0.72, 39.03 |  |
| Female |  |  |  |  | < 0.01 |
| < 5 months | 229 | 1,732.4 | Ref. | Ref. |  |
| 5–14 months | 739 | 6,563.7 | 1.54 | 0.18, 12.80 |  |
| 15–29 months | 749 | 7,295.8 | 2.53 | 0.33, 19.55 |  |
| ≥ 30 months | 1036 | 12,981.6 | 3.98 | 0.54, 29.41 |  |
|  |  |  |  |  |  |
| 20–49 years |  |  |  |  | < 0.01 |
| < 5 months | 177 | 2,342.8 | Ref. | Ref. |  |
| 5–14 months | 670 | 9,321.0 | 1.68 | 0.21, 13.75 |  |
| 15–29 months | 651 | 10,031.2 | 1.85 | 0.23, 14.67 |  |
| ≥ 30 months | 947 | 17,125.0 | 5.22 | 0.71, 38.39 |  |
| ≥ 50 years |  |  |  |  | 0.02 |
| < 5 months | 63 | 699.6 | Ref. | Ref. |  |
| 5–14 months | 239 | 2,894.4 | 1.98 | 0.24, 16.17 |  |
| 15–29 months | 283 | 3,487.5 | 3.06 | 0.40, 23.29 |  |
| ≥ 30 months | 575 | 8,064.4 | 3.59 | 0.49, 26.40 |  |
|  |  |  |  |  |  |
| High school or lower |  |  |  |  | < 0.01 |
| < 5 months | 290 | 1,112.8 | Ref. | Ref. |  |
| 5–14 months | 1009 | 5,180.2 | 2.32 | 0.30, 18.19 |  |
| 15–29 months | 1021 | 5,936.0 | 2.87 | 0.38, 21.78 |  |
| ≥ 30 months | 1384 | 12,747.8 | 4.51 | 0.62, 32.91 |  |
| College or higher |  |  |  |  | < 0.01 |
| < 5 months | 156 | 1,929.7 | Ref. | Ref. |  |
| 5–14 months | 534 | 7,035.2 | 1.23 | 0.14, 11.01 |  |
| 15–29 months | 525 | 7,582.8 | 2.15 | 0.27, 17.31 |  |
| ≥ 30 months | 758 | 12,441.6 | 5.46 | 0.73, 40.85 |  |

Abbreviations: HR, hazard ratio; CI, confidence interval; Ref., reference.

The results were estimated from Cox proportional hazards models adjusted for sex, age at initial exposure, educational level, tobacco smoking, and distance from humidifier.
