# Supplementary material for: Association between humidifier disinfectant use duration and lung cancer development in Korea
Source: Epidemiol Health. 2025 May 2;47:e2025023. doi: 10.4178/epih.e2025023 (PMC12425697; doi:10.4178/epih.e2025023)
Supplement: Supplementary Material 2. [file epih-47-e2025023-Supplementary-2.docx]

*Supplementary Material 2*

**Association between humidifier disinfectant use duration and lung cancer development in the Republic of Korea**

Sungchan Kang, Jeong-In Hwang**,** Su Hwan Kim, Hyungryul Lim, Dong-wook Lee, Woojoo Lee, Jong Hun Kim, Sol Yu, Jungyun Lim, Younghee Kim, Kyoung-Nam Kim

**Table of Contents**

**Table S2.** Hazard ratios and 95% confidence intervals for lung cancer occurrence based on duration of humidifier disinfectant use, estimated using latency periods of 6 and 8 years

**Table S2.** Hazard ratios and 95% confidence intervals for lung cancer occurrence based on duration of humidifier disinfectant use, estimated using latency periods of 6 and 8 years

| Duration | *n* | Person-year | | Hazard ratio | 95% CI | *p*-value for trend |
| --- | --- | --- | --- | --- | --- | --- |
| Latency period of 6 years | | |  | | | |
| Categorical variable | |  | |  |  | < 0.01 |
| < 5 months | 234 | 3,013.58 | | Ref. | Ref. |  |
| 5–14 months | 888 | 12,107.01 | | 1.13 | 0.24, 5.24 |  |
| 15–29 months | 888 | 13,302.41 | | 1.65 | 0.38, 7.26 |  |
| ≥ 30 months | 1,439 | 24,781.78 | | 3.95 | 0.95, 16.37 |  |
| Continuous variable | |  | |  |  |  |
| Per one month | 3,449 | 53,204.77 | | 1.01 | 1.01, 1.02 | < 0.01 |
|  |  |  | |  |  |  |
| Latency period of 8 years | | |  | | | |
| Categorical variable | |  | |  |  | < 0.01 |
| < 5 months | 225 | 2,948.69 | | Ref. | Ref. |  |
| 5–14 months | 868 | 11,968.38 | | 0.84 | 0.17, 4.06 |  |
| 15–29 months | 860 | 13,106.91 | | 1.18 | 0.26, 5.37 |  |
| ≥ 30 months | 1,356 | 24,198.89 | | 2.94 | 0.7, 12.32 |  |
| Continuous variable | |  | |  |  |  |
| Per one month | 3,309 | 52,222.86 | | 1.01 | 1.01, 1.02 | < 0.01 |

Abbreviations: CI, confidence interval; Ref., reference.

The results were estimated from Cox proportional hazards models adjusted for sex, age at initial exposure, educational level, tobacco smoking, and distance from humidifier.
